# Functional connectivity changes in cerebral small vessel disease - a systematic review of the resting-state MRI literature

**DOI:** 10.1186/s12916-021-01962-1

**Published:** 2021-05-05

**Authors:** Maximilian Schulz, Caroline Malherbe, Bastian Cheng, Götz Thomalla, Eckhard Schlemm

**Affiliations:** 1 Department of Neurology, University Medical Centre Hamburg-Eppendorf, Hamburg, Germany; 2 Department of Computational Neuroscience, University Medical Centre Hamburg-Eppendorf, Hamburg, Germany

**Keywords:** Brain network, Cerebral small vessel disease, Cognition, Functional connectivity, Magnetic resonance imaging, Resting state, Risk of bias, Patho-connectomics, Systematic review

## Abstract

**Background:**

Cerebral small vessel disease (CSVD) is a common neurological disease present in the ageing population that is associated with an increased risk of dementia and stroke. Damage to white matter tracts compromises the substrate for interneuronal connectivity. Analysing resting-state functional magnetic resonance imaging (fMRI) can reveal dysfunctional patterns of brain connectivity and contribute to explaining the pathophysiology of clinical phenotypes in CSVD.

**Materials and methods:**

This systematic review provides an overview of methods and results of recent resting-state functional MRI studies in patients with CSVD. Following the Preferred Reporting Items for Systematic Reviews and Meta-Analysis (PRISMA) protocol, a systematic search of the literature was performed.

**Results:**

Of 493 studies that were screened, 44 reports were identified that investigated resting-state fMRI connectivity in the context of cerebral small vessel disease. The risk of bias and heterogeneity of results were moderate to high. Patterns associated with CSVD included disturbed connectivity within and between intrinsic brain networks, in particular the default mode, dorsal attention, frontoparietal control, and salience networks; decoupling of neuronal activity along an anterior–posterior axis; and increases in functional connectivity in the early stage of the disease.

**Conclusion:**

The recent literature provides further evidence for a functional disconnection model of cognitive impairment in CSVD. We suggest that the salience network might play a hitherto underappreciated role in this model. Low quality of evidence and the lack of preregistered multi-centre studies remain challenges to be overcome in the future.

## Background

Cerebral small vessel disease (CSVD) is a term that describes clinical, neuroimaging, and pathological features assumed to arise from compromised blood flow in the intrinsic cerebral arteriolar system [1]. In its later stages, CSVD is associated with neurological symptoms, in particular lacunar ischaemic stroke, and cognitive impairment ranging from mild deficits to vascular dementia [2, 3]. Small vessel disease is estimated to be the main etiological factor in up to 23% of all ischaemic strokes [4] and to be the second most common contributing factor to dementia after Alzheimer’s pathology [5] and is thus responsible for a growing disease burden in ageing societies.

Even in its pre-symptomatic stage, CSVD is associated with structural brain changes on neuroimaging, in particular white matter hyperintensities (WMH) of presumed vascular origin, lacunes, cerebral microbleeds, enlarged perivascular spaces, and brain atrophy [6]. Cardiovascular risk factors, such as hypertension, diabetes, smoking, or dyslipidaemia, are associated with both WMH and the clinical sequelae associated with CSVD [7, 8].

In recent years, the network perspective on the human brain has revolutionised neuroscience and advanced our understanding of neurological and psychiatric disorders [9–12]. The network paradigm posits that different brain regions, while spatially remote, are structurally and functionally linked and interact to facilitate brain functions. Analysis of structural brain networks by magnetic resonance diffusion tensor imaging revealed that WMH disrupts the topological organisation of the brain connectome and that the associated loss of network efficiency links vascular risk burden and cognitive impairment [13–16]. Nevertheless, there remains considerable variability in clinical phenotypes, such as cognitive impairment or affective functions, that is not explained by structural markers alone [17–19].

Functional connectivity (FC), on the other hand, is defined as the pattern of synchronous neuronal activation [20], which, in turn, can be probed in vivo using the blood-oxygen level-dependent (BOLD) signal in magnetic resonance imaging (MRI) [21]. Functional connectivity can be analysed either in response to tasks and external stimuli or in the resting-state which minimises the cognitive and behavioural demand on subjects [21]. The latter provides a description of the spatiotemporal organisation of brain activity, from which discrete modes can be extracted as intrinsic resting-state networks that correspond to specific cognitive domains [22].

Recently, the benefits of such a shift of perspective toward a more global understanding of brain function have also been recognised for cerebral small vessel disease [23]. While the clinical benefits of understanding patterns of disrupted FC associated with CSVD might seem, at the moment, very limited, our vision is that, ultimately, it might contribute to designing and implementing patient-specific interventions in the form of neuropsychological training or electromagnetic stimulation to help ameliorate cognitive impairment. Evidence for the relevance of disturbed connectivity especially in the default mode, dorsal attention, and frontoparietal control networks to cognitive impairment in CSVD has been reviewed previously, covering the literature up to 2014 [24]. In the present article, we provide an overview over the rapidly expanding recent literature on altered resting-state connectivity patterns associated with CSVD. In contrast to previous work, we include studies of both clinically healthy individuals and patients with manifest CSVD and consider both distributed networks and point-to-point connectivity. In order to keep the review focused, we restrict attention to resting-state functional MRI studies and do not review studies using a task-based design or different imaging modalities, such as electro- or magnetoencephalography. The goal is to take stock of the current literature, review methodological advances in recent years, and update our understanding of the neural mechanisms underlying the cognitive deficits that patients with CSVD face.

## Methods

A systematic review of the literature was performed according to the Preferred Reporting Items for Systematic Reviews and Meta-Analysis (PRISMA) statement [25]; the protocol for the review was not preregistered.

### Literature search and study selection

Inclusion criteria for articles considered in this review were as follows: (1) written in English, (2) analysing exclusively human study participants, (3) published after January 2010, (4) radiological evidence of sporadic cerebral small vessel disease with structural brain imaging showing manifestations of CSVD in the form of white matter hyperintensities in at least a subset of the study population, and (5) analysis of resting-state functional connectivity using functional MRI. We excluded review articles; descriptions of ongoing studies; functional imaging studies using only electroencephalography, magnetoencephalography, or positron emission tomography; and reports concentrating exclusively on patients with non-sporadic CSVD, e.g. of genetic origin, or non-vascular dementias, e.g. Alzheimer’s disease.

Following a prespecified search strategy, the PubMed online database was queried for studies published between January 2010 and November 2020 using the conjunction of keywords specific for pathology (‘small vessel disease’, ‘white matter lesion’, ‘leukoaraiosis’, ‘microangiopathy’), network science (‘connectivity’, ‘network’, ‘graph’, ‘module’), and imaging modality (‘MRI’, ‘BOLD’, ‘resting state’) as search criteria (see Additional file 1 for the exact search strategy). In addition, references of search results were screened for further eligible articles. Studies were discarded if the title or abstract indicated failure to meet all the specified inclusion or satisfaction of at least one exclusion criterion. The remaining articles were read in full and evaluated according to the stated criteria.

The risk of methodological bias in individual studies (PRISMA items 12 and 19) was assessed using the Appraisal tool for Cross-Sectional Studies (AXIS tool) [26], modified to not contain items related to presentation of the Results, the Discussion of findings, or the Funding of the study [27]. Detailed descriptions of individual items are presented in Additional file 1: Table S1. Based on the number of quality criteria satisfied, each study was assigned an integer score from 0 (no criteria satisfied) to 11 (all criteria satisfied). Trichotomising this ordinal scale, we classified the risk of bias as high (score 0–3), moderate (4–7), or low (8–11). We strived to cover the literature comprehensively, and even a high risk of basis was therefore not defined as an exclusion criterion for this review.

### Data extraction and analysis

After screening, the following data were extracted from the articles: year of publication; sample size; average age and clinical characteristics of study populations including measures undertaken to minimise confounding by comorbidities; the employed operationalisation of cerebral small vessel disease and severity grading of WMH; details of the scanning parameters and pre-processing steps including the controversial topics of motion scrubbing and global signal regression; the analytical approach to functional connectivity; and key results regarding patterns of altered connectivity in patients with CSVD and, if reported, their relation to cognitive performance.

For ease of presentation, studies were classified according to clinical characteristics of the study population—manifest CSVD, healthy participants, or others, not primarily vascular clinical conditions—and their main approach to quantifying and analysing connectivity. These predefined analytical categories included short-range connectivity within a part of the brain, long-range connectivity between pairs of remote brain areas defined either a priori or using a data-driven approach, and global analyses of topological properties of the functional connectome. We also reviewed the cognitive tests applied in these studies and associations of cognitive ability with functional connectivity measurements.

## Results

### Study characteristics

**Fig. 1:**
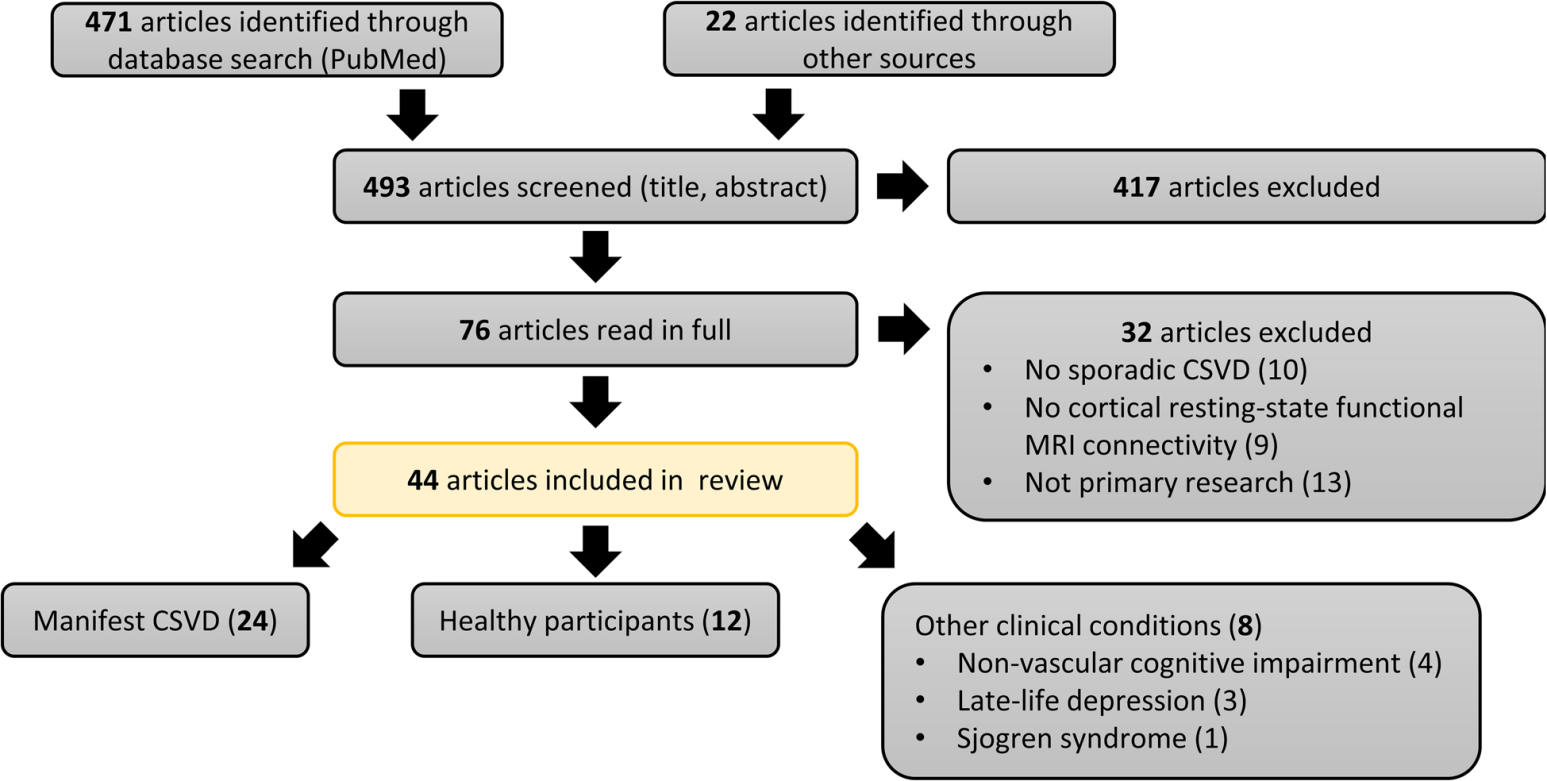
Systematic literature search and article screening results. The PubMed electronic database was searched on 1 December 2019, on 28 June 2020, and again on 22 November 2020. Together with articles obtained through other sources (personal communication, cited articles), 493 papers were identified, screened by M. S., and assessed based on inclusion and exclusion criteria. Full texts were obtained for 76 articles; these were assessed against the stated criteria by M. S. and E. S. The included 44 articles were classified on the basis of characteristics of the study population

The results of the search and selection process are summarised in Fig. 1. We identified a total of 493 potentially relevant papers, 471 of which were obtained by searching PubMed and 22 through personal communication or as references cited in other works. Four hundred seventeen papers were excluded based on their title or abstract. Of the remaining 76 studies, which were read in full by both MS and ES, 44 were included in this review. Details of individual studies are summarised in Table 1.

**Table 1:** Summary of included articles analysing resting-state functional connectivity in patients with manifest cerebral small vessel disease (CSVD). We report imaging and clinical characteristics of patients included in each study, key steps in the acquisition and pre-processing of BOLD data, analysis of functional connectivity, and FC patterns found to be associated with CSVD. Descriptive statistics as extracted from articles are reported as mean ± standard deviation. Missing information is indicted by empty brackets ([]). Arrows indicate increased (↑) or reduced (↓) values, as well as positive (↗) and negative (↘) associations

Reference	Participants	Characteristics of patients with CSVD		MRI acquisition params	BOLD pre-processing	FC analysis	FC patterns associated with CSVD	Risk of bias (AXIS)
		Neuroimaging	Cognition					
[28]	18 CSVD-CN 16 CSVD-MCI	WMH and lacunes (clinical diagnosis)	*MMSE* CN 28.9 ± 1.3 MCI 28.1 ± 1.4	Philips Achieva, 3 T TR 2000 ms, TE 30 ms 64 × 64 × [] 3.6 × 3.6 × 4 mm^3^ 220 volumes eyes open	DPARSF v2.0, SPM 5, REST [Confound regression] Motion scrubbing - Max motion > 1 mm/1°	PCC from external template SCA to define DMN Pearson correlation	↓ FC in DMN in the left middle temporal gyrus, left ant. cingulate/left middle frontal gyrus, right caudate, right middle frontal gyrus, and left medial frontal gyrus/paracentral lobule ↑ FC in DMN in the right inf. temporal gyrus, left middle temporal gyrus, left precentral gyrus, and left sup. parietal lobule	High (2)
[29]	29 CSVD	WMH (clinical diagnosis) *WMH volume* VRF 0, 20.4 ± 19.3 VRF 1, 27.3 ± 21.5 VRF 3, 17.4 ± 19.5	*MoCA* VRF 0, 25.8 ± 3.5 VRF 1, 22.0 ± 6.2 VRF 3, 21.0 ± 5.4	Philips Achieva, 3 T TR 2000 ms, TE 30 ms 64 × 64 × 40 3.6 × 2.9 × 3 mm^3^ 184–259 volumes, [eyes]	FSL v4.1 Confound regression - ICA Motion scrubbing - ‘relative motion’ > 1.5 mm	ICA to define DMN, SMN, medial-visual RSN Pearson correlation	FC in DMN ↗ cardiovascular reactivity	Moderate (4)
[30]	21 CSVD-CN 16 CSVD-MCI 13 SVaD	WMH (clinical diagnosis)	*MMSE* CN 28.9 ± 0.7 MCI 26.3 ± 1.0 SVaD 20.5 ± 2.3 *MoCA* CN 24.6 ± 2.9 MCI 20.6 ± 2.8 SVaD 15.1 ± 3.6	Siemens, 3 T TR 2480 ms, TE 30 ms 64 × 64 × 36, 3 × 3 × 4 mm^3^ 240 volumes eyes closed	SPM 8 [Confound regression] Motion scrubbing - Head motion > 2 mm/2°	AAL atlas Pearson correlation, (extended) maximal information coefficient SVM classification	None	High (3)
[31]	46 CSVD 41 CADASIL	*WMH volume* 0.3% [0.03, 3.1]% of intracranial volume *Lacunes* 0 (0) [0–20]	MMSE median 29, IQR [26, 32]	Siemens Skyra/Prisma, 3 T, 64-/32-channel head coils, multi-band × 8 TR 700 ms, TE 39 ms [matrix] 3 × 3 × 3 mm^3^ 2.4 × 2.4 × 2.4 mm^3^ 675–700 volumes eyes closed	FSL, ANTs, BRAMILA v2.0 Confound regression - ICA-AROMA non-aggressive - Linear trend, 6 motion params - GSR: CSF, WM Motion scrubbing - Volume censoring (FD > 0.5 mm, total time < 5 min)	Power atlas to define DMN, FPCN, hand-SMN, VN Pearson correlation graph theory	No association between WMH and FC Poor reproducibility of network measures in CSVD	Moderate (6)
[33]	33 MCI with confluent (C) WMH 30 MCI with non-confluent (NC) WMH	*Fazekas* C-WMH 8.0 ± 2.4 NC-WMH 3.2 ± 1.2 *WMH volume* C-WMH 8.7 ± 9.2 ml NC-WMH 1.9 ± 1.5 ml	*MMSE* C-WMH 27.6 ± 1.5 NC-WMH 27.8 ± 1.7	Siemens Trio, 3 T TR 2000 ms, TE 30 ms [] × []× 36, 3 × 3 × 3 mm^3^ 240 volumes [eyes]	CONN 18b Confound regression - GSR: WM, CSF - Motion parameters Motion correction - Artefact Detection Tools - Spike regression (FD > 0.5 mm)	CONN atlas with 138 ROIs to define DMN, SMN, VN, DAN, FPCN language, and cerebellar networks Bivariate correlation coefficients	↑ Interregional FC (C-WMH > NC-WMH) ↑ Intra-regional FC (C-WMH > NC-WMH) Few ↓ interregional FC (C-WMH < NC-WMH)	High (2)
[34]	29 CSVD with gait disorder (GD+) 29 CSVD without gait disorder (GD−)	Fazekas ≥2 *WMH volume* GD+, 6.12 mm^3^ GD−, 3.53 mm^3^ *Lacunes* GD+, 7.45 ± 4.01 GD−, 5.62 ± 3.35	*MMSE* GD+, 25.14 ± 3.24 GD−, 25.69 ± 3.32	GE Signa, 3 T, 8-channel head coil TR 2000 ms, TE 30 ms 64 × 64 × [] 3.75 × 3.75 × 4.6 mm^3^ 240 volumes eyes closed	DPABI, SPM 8 Confound regression - Linear trend, 24p - GSR: CSF, WM Motion scrubbing - ‘displacement’ > 2.5 mm /2.5°	fALFF Pearson correlation VBM	↓ FC between left SMA and temporal lobe (GC+ < GD−) ↗ gait speed fALFF in left SMA ↗ cadence	Moderate (4)
Healthy controls and patients with CSVD without mandatory cognitive impairment								
[35]	12 CSVD 21 HC	Wahlund 8.3 ± 4.0 Lacunes 1.9 ± 2.4	MMSE 27.6 ± 1.5	[Scanner, field str.] TR 2300 ms, [TE] [matrix], 3 × 3 × 4 mm^3^ 300 volumes [eyes]	FSL, AFNI, SPM B_0_ field map correction Confound regression - Linear/quadratic trend - 6 motion parameters - GSR: WM, CSF signal [Motion scrubbing]	Eigenvector centrality	↓ FC in ventromedial PFC, MCC, and sup. parietal lobe ↑ FC in the cerebellar regions I–VI FC ↗ WMH in the middle temporal sulcus, inf. temporal gyrus, cerebellar lobules Crus II, VIIb, VIII FC ↘ WMH in ventromedial PFC, SMA, PCC, and sup. parietal lobe	High (1)
[36]	30 CSVD 26 HC	Fazekas 1–3 Lacunes excluded	MoCA 22.9 ± 3.4 (pooled)	Siemens, 3 T TR 2480 ms, TE 30 ms 64 × 64 × 36, 3 × 3 × 4 mm^3^ 240 volumes, eyes closed	SPM 8 [Confound regression] Motion scrubbing - Head movement > 2 mm/2°	ICA to define SMN voxel-wise two-sample *t* test	↓ FC in the right cingulate motor area, left posterior insula, and left ventral premotor area	High (2)
[37]	28 CSVD 30 HC	Fazekas 2.9 ± 1.2	MMSE 27.9 ± 1.6 MoCA 25.7 ± 2.0	Siemens Trio, 3 T TR 2000 ms, TE 30 ms 64 × 64 × [] 3.5 × 3.5 × 3 mm^3^ 240 volumes [eyes]	SPM 8, DPABI, REST [Confound regression] Motion scrubbing - Head movement > 2 mm/2°	ALFF, SCA	↓ ALFF in the left parahippocampus ↑ ALFF in the left inf. semi-lunar lobule and right frontal sup. orbital gyrus ↑ FC between right insula–right sup. orbitofrontal gyrus and right calcarine–left parahippoca. gyrus	High (2)
[38]	16 CSVD 13 HC	WMH (clinical diagnosis, Fazekas)	MMSE 23.7 ± 3.9 MoCA 18.3 ± 4.1	Siemens, 3 T TR 2000 ms, TE 30 ms 64 × 64 × 35 3 × 3 × 3 mm^3^ 230 volumes eyes closed	SPM 8 Confound regression - Linear trend Motion scrubbing - Head movement > 2 mm/2°	Regional homogeneity	↓ ReHo in the left insula, right sup. temporal gyrus, Rolandic operculum, precentral gyrus, and cerebellum; bilateral ant. cingulate gyrus, ant. MCC, PFC, and SMA ↑ ReHo values in the left middle temporal gyrus, cuneus and sup. occipital gyrus, and the bilateral angular gyrus, precuneus, postcentral gyrus, inf. and sup. parietal gyrus	Moderate (5)
[39]	15 CSVD 15 HC	WMH (clinical diagnosis, Fazekas)	MMSE 23.7 ± 4.0 MoCA 18.6 ± 4.1	Siemens, 3 T TR 2000 ms, TE 30 ms 64 × 64 × [] 3 × 3 × 4 mm^3^ 230 volumes eyes closed	DPABI v2.1 Confound regression - Linear trend - 24p [40] - GSR: WM, CSF - Spike regressor (FD > 0.2 mm) Motion scrubbing - Mean FD > group mean + 2*SD	36 ROIs representing DMN, DAN, FPCN, SMN, VN Pearson correlation Network-based statistics (NBS) [41]	↓ AN-SMN network FC ↑ DAN-FPCN network FC ↓ Edge FC SMN-AN, SMN-VN, FPCN-AN and DAN-VN pairs, and within AN and VN ↑ Edge FC in DMN-AN, DMN-FPCN, and DAN-FPCN pairs	High (3)
[42]	26 lacunar stroke patients 19 HC	Fazekas ≥2 Lacunes 1–17 (6) WMH 33 ± 34 ml	History of lacunar stroke syndrome	Siemens Verio, 3 T, 32-channel head coil TR 2430 ms TE 1/2/3 13/31/48 ms 64 × 64 × 34 3.75 × 3.75 × 4.18 mm^3^ 269 volumes, eyes open	SPM, CONN Confound regression - Motion parameters [details] - GSR: WM, CSF [Motion scrubbing]	Desikan–Killiany parcellation Pearson correlation Probab. tractography Graph theory	No difference in FC or brain network topology	Moderate (6)
[43]	36 CSVD 31 HC	Fazekas ≥2	*MMSE* HC 28.4 ± 1.8 CSVD 26.4 ± 3.7	Siemens Verio, 3 T, 12-channel head coil TR 2000 ms, TE 25 ms 64 × 64 × [z] 3.75 × 3.75 × 4 mm^3^ [volumes], [eyes]	DPARSF, SPM 8 Confound regression - 6 head motions params - GSR: global, WM, CSF Motion scrubbing - Head movement > 2 mm/2°	AAL 90 Pearson correlation Graph theory NBS	↓ Global FC ↑ Characteristic path length ↓ Global efficiency	High (0)
Healthy controls and patients with CSVD with mandatory cognitive impairment								
[44]	26 svMCI 28 HC	Wahlund ≥ 2 ± lacunes	Subjective complaints MMSE 25.7 ± 2.7	Siemens, 3 T TR 2000 ms, TE 40 ms 64 × 64 × 28 4 × 4 × 5 mm^3^ 239 volumes eyes closed	SPM 5 Confound regression - Linear trend - 6 motion profiles - GSR: WM, CSF Motion scrubbing - Head movement > 3 mm/3°	ALFF, SCA, FC density Pearson correlation (only positive)	↓ ALFF in bilateral medial PFC ↑ ALFF in right PCC, precuneus, right hippocampus, right thalamus ↓ FC in DMN (PCC/precuneus, medial PFC, sup. frontal gyrus, inf. parietal lobule and hippocampus) and inferior/middle frontal gyrus	High (2)
[45]	21 svMCI 26 HC	Wahlund ≥ 2 ± lacunes	Subjective complaints MMSE 25.5 ± 2.7	Siemens, 3 T TR 2000 ms, TE 40 ms 64 × 64 × 28 4 × 4 × 5 mm^3^ 239 volumes eyes closed	SPM 5 and 8, REST, DARTEL Confound regression - Linear trend - 6 motion profiles - GSR: WM, CSF Motion scrubbing - Head movement > 3 mm/3° - Volume censoring (FD > 0.3 mm, < 3 min)	H-1024 parcellation Pearson correlation Graph theory	↑ Characteristic path length, ↑ modularity ↑ Within-module degree in the medial PFC, left insula, and cuneus ↓ Within-module degree in the middle cingulate gyrus ↑ Participation coefficient in the left inferior/superior parietal cortex	High (1)
[46]	37 PiB+ AD 37 PiB− SVaD 13 mixed dementia 65 HC	Fazekas (severe)	*MMSE* PiB+ AD, 18.1 ± 4.2 PiB− SVaD, 21.5 ± 4.3 MD, 17.9 ± 4.9 HC, 28.9 ± 1.2	Philips Achieva, 3 T, 8-channel head coil TR 3000 ms, TE 35 ms [matrix], 1.7 × 1.7 × 4 mm^3^ 100 volumes, eyes open	AFNI Despiking [Confound regression] Motion scrubbing - Max FD > 0.3 mm - Head movement > 2 mm/2°	ICA to define DMN and FPCN W-score maps	↓ FC in DMN in the left superior frontal gyrus ↓ FC in FPCN in the left insula	Moderate (5)
[47]	32 CSVD-CI 23 HC	Fazekas 2–3 ± lacunes	MMSE 23.8 ± 2.7	GE Signa, 3 T TR 2000 ms, TE 30 ms 64 × 64 × [] 3.75 × 3.75 × 4.6 mm^3^ 240 volumes eyes closed	SPM 8, DPARSF, CONN Confound regression - compCor - GSR: global signal [Motion scrubbing]	Medial PFC and thalamus from ext. template SCA Pearson correlation	↓ FC between the left thalamus and right sup. temporal gyrus, left sup. frontal gyrus, left and putamen ↓ FC between the right thalamus and left inferior temporal gyrus ↑ FC between the bilateral thalamus and right inferior/middle frontal gyri ↓ FC between the med. PFC and bilateral SMA, thalamus, left sup. frontal gyrus, ACC, sup. parietal lobe, and hippocampus	High (2)
[48]	14 CSVD-CN 27 CSVD-MCI 12 SVaD 30 HC	Fazekas (cut-off not specified)	*MMSE* CSVD-CN 29.1 ± 1.0 CSVD-MCI 27.2 ± 2.4 SVaD 23.3 ± 3.4	Siemens, 3 T TR 2000 ms, TE 30 ms 64 × 64 × 32, 4 × 4 × 3.7 mm^3^ [volumes], eyes closed	SPM 8 [Confound regression] Motion scrubbing - Head movement > 2 mm/2°	ICA to define DMN, SN, FPCN SCA from the right fronto-insular cortex	↓ FC between SN-DMN ↑ FC between SN-FPCN and within SN	High (3)
[49]	36 CSVD 50 AD 55 HC	*WMH volume* svMCI 22.6 ± 6.1 ml SVaD 44.8 ± 10.1 ml *Lacunes* svMCI 6.5 ± 7.5 SVaD 7.4 ± 7.2 *Microbleeds* svMCI 13.4 ± 18.7 SVaD 26.0 ± 49.2	*MMSE* svMCI 25.4 ± 3.7 SVaD 17.4 ± 4.2	Siemens, 3 T TR 3000 ms, TE 35 ms [matrix] 3.4 × 3.4 × 3.4 mm^3^ 200 volumes [eyes]	SPM 12 No smoothing Confound regression - Motion parameters - Linear trend - GSR: WM, CSF Motion scrubbing - Volume censoring (FD > 1 mm, > 30%)	Schaefer 400 atlas Pearson correlation AV1451 tau-PET	FC ↗ tau covariance, no associations with WMH	Moderate (7)
[50]	25 CSVD-CN 24 CSVD-MCI 36 HC	Fazekas ≥2 ± lacunes WMH CN 3.0 (0.76, 4.0) ml MCI 4.8 (0.76, 5.6) ml	*MMSE* CN 28.4 ± 1.3 MCI 27.8 ± 2.1 *MoCA* CN 26.1 ± 0.5 MCI 21.4 ± 0.5	Philips, 3 T TR 2000 ms, TE 30 ms 64 × 64 × 35 3.75 × 3.75 × 4 mm^3^ 230 volumes eyes closed	GRETNA v2.0, SPM 8 Confound regression - Linear trend, 24p - GSR: GS, WM, CSF Motion scrubbing - Head movement > 2 mm/2°	H-1024 parcellation Pearson correlation Graph theory to define DMN, FPCN, SMN, and VN	No robust associations	Moderate (4)
[51]	21 CSVD-CN 20 CSVD-MCI 25 HC	WMH volume CN 3.2 ± 3.0 ml CI 3.4 ± 4.1 ml ± lacunes	*MMSE* CN 28.5 ± 1.3 MCI 28.1 ± 1.3 *MoCA* CN 25.6 ± 1.9 MCI 22.2 ± 2.9	Philips, 3 T, 32-channel head coil TR 2000 ms, TE 30 ms 64 × 64 × 35 3.75 × 3.75 × 4 mm^3^ 230 volumes Eyes closed	DPABI Confound regression - 24p - GSR: GS, WM, CSF Motion scrubbing - Head movement > 3 mm/3°	PCC, dlPFC, IPS from external template SCA to define DMN, FPCN, and DAN	↓ FC in DMN in the right thalamus, hippocampus, and precuneus ↑ FC in FPCN in the right inf. parietal lobule ↓ FC between PCC-FPCN in the left precentral gyrus and bilateral middle cingulate gyri ↓ FC between PCC-DAN in the bilateral paracentral lobule and precuneus ↓ FC between PCC-DAN in the bilateral PCC and right precuneus	Moderate (6)
[52]	14 CSVD + THA LAC 27 CSVD − THA LAC 34 HC	Fazekas 3–6 *Lacunes* LAC+, 5.6 ± 3.9 LAC−, 2.0 ± 1.4	*MMSE* LAC+, 26.3 ± 2.8 LAC−, 28.1 ± 2.0	GE Discovery, 3 T TR 2000 ms, TE 35 ms 64 × 64 × 36 3.4 × 3.4 × 4 mm^3^ 210 volumes eyes closed	SPM 8 Confound regression - 6 motion parameters - GSR: WM, CSF Motion scrubbing - Head movement > 2 mm/2°	AAL 90 Pearson correlation Graph theory, Network-based statistics	↓ FC in para-/limbic and subcortical regions	Moderate (5)
[53]	32 CSVD-MCI 20 SVaD 35 HC	Fazekas ≥1	*MMSE* MCI 25.5 ± 1.8 SVaD 22.0 ± 2.0 *MoCA* MCI 22.2 ± 1. SVaD 17.4 ± 2.9	Siemens Verio, 3 T TR 2000 ms, TE 30 ms 64 × 64 × 32 4 × 4 × 4.2 mm^3^ 240 volumes eyes closed	DPARSF Confound regression - Linear/quadratic, 24p - GSR: WM, CSF Motion scrubbing - Head movement > 3 mm/3°	AAL atlas Pearson correlation Graph theory	↓ Global FC ↓ Small-worldness	Moderate (4)
[54]	20 LA-VAD 32 LA-VCIND 35 HC	Not reported	*MMSE* HC 29.4 ± 1.1 LA-VCIND 27.9 ± 1.8 LA-VaD 23.0 ± 3.8 *MoCA* HC 26.0 ± 2.5 LA-VCIND 25.8 ± 2.0 LA-VaD 23.2 ± 2.8	Siemens, 3 T TR 2000 ms, TE 30 ms 64 × 64 × 20 4 × 4 × 6 mm 250 volumes eyes closed	DPARSF Confound regression - 24p - GSR: WM, CSF Motion scrubbing - Head movement > 3 mm/3°	Group ICA (GIFT) Granger causality	Unclear; text and figures not consistent	High (2)

The number of subjects, including both patients with small vessel disease and controls depending on study design, varied between 11 and 1584 with a median sample size of 72.5 (interquartile range [IQR] 50.8–106.8). Mean age across studies ranged from 50.0 to 76.4 years, with a median of 66.0 years (IQR 62.4–69.8 years).

Regarding the underlying research questions, roughly half of the included studies (24/44) reported the investigation of altered functional connectivity patterns in the presence of cerebral small vessel disease and its relation to cognitive ability as their primary research objective. Of these, six reports focused on patients with CSVD exclusively, whereas the study designs of the remaining reports involved comparing groups of healthy controls, patients with non-vascular cognitive impairment, or both. Twelve studies reported measures of cerebral small vessel disease, often as part of a more comprehensive assessment of structural brain parameters, and functional connectivity in populations of healthy participants without clinically manifest vascular pathology or cognitive impairment. Eight articles addressed functional connectivity in the context of other clinical conditions not directly related to vascular pathology, such as tau pathology-associated cognitive impairment or depression, but included markers of small vessel disease as covariates.

### Operationalisation of CSVD and associated cognitive impairment

All of the 24 MRI studies reporting on resting-state functional connectivity in the context of clinically overt CSVD defined the presence of white matter hyperintensities on T2-weighted cerebral MR imaging as one of their inclusion criteria. In more than half of the studies (14/24), these were evaluated according to the ordinal Fazekas scale [55, 56]; in three studies, authors chose the Wahlund scale to assess age-related white matter changes [32]; white matter lesion load was also quantified volumetrically in eight studies; no precise definition of imaging criteria was reported in five articles.

When white matter disease was reported as a structural covariate in the investigation of functional connectivity, the extent of structural changes was quantified using either absolute or relative white matter hyperintensity volumes. Techniques for segmenting WMH on either T2 or FLAIR sequences included manual, semi-manual, and fully automated approaches; in one case, the algorithm was not described [57].

Beyond the presence of white matter lesions, evidence of lacunes or recent lacunar infarcts was considered in 13/24 studies; the distinction between the two entities was often imprecise, with only three articles referring to the STandards for ReportIng Vascular changes on nEuroimaging (STRIVE, [6]) consensus statement in this context [31, 50, 51]. Reflecting their conceptualisation as fluid-filled cavities, lacunes were defined as hypointense ovoid regions on T2- or FLAIR-weighted imaging with a diameter ranging from [2–3] to [15–20] mm. Three studies required patients with CSVD to have evidence of at least one lacuna or recent lacunar infarct [28, 42, 52], while one report excluded such patients [36]. Information on the number of lacunes contributed to the definition of a compositive CSVD score in one study [49]; in the remaining cases, it was either reported descriptively or used as a covariate in statistical analyses [50]. While most studies specified cortical or large subcortical infarcts as an exclusion criterion, one article included such patients specifically [43].

In addition to imaging findings, clinical characteristics were used to define patient cohorts. This was done to either separate patients and participants with and without cognitive impairment; to differentiate patients with CSVD from patients with non-vascular cognitive impairment, especially Alzheimer’s disease; or to grade the severity of vascular cognitive impairment, ranging from cognitively normal (CN) over mildly affected (variably called *subcortical vascular mild cognitive impairment* [svMCI], or *vascular cognitive impairment no dementia* [VICND]) to subcortical vascular dementia (SVaD). In addition to dedicated diagnostic criteria for different dementias [58, 59], cognitive assessment was based predominantly on scales such as the Mini Mental State Examination (MMSE), Montreal Cognitive Assessment battery (MoCA), or Clinical Dementia Rating scale (CDR). A minority of studies used the Petersen criteria and included functional activities and temporal evolution of cognitive abilities in their definition of mild cognitive impairment [60, 61]. Only three studies employed positron emission tomography (PET) to distinguish tau and/or amyloid pathology from purely vascular disease [49, 62, 63], and the risk of confounding by mixed disease seems therefore high in the majority of reported studies.

### Functional MRI acquisition and pre-processing

Magnetic resonance imaging was performed on scanners from a variety of vendors (Siemens, Philips, GE), usually at 3 Tesla. The use of specialised receiver head coils, multi-band, or multi-echo techniques was rarely reported. Repetition time (TR) and echo time (TE) were predominantly set at 2000 ms and 30 ms, respectively, with exceptional values ranging from 700 to 4500 ms and 13 to 84 ms, respectively. Reconstructed voxel sizes in the BOLD scans varied in the range [1.7–4] × [1.7–4] × [2–6] mm^3^, arranged in a three-dimensional matrix of dimensions varying in the range [64–128] × [64–128] × [20–64]. The number of acquired BOLD volumes varied between 100 and 700 (median 230, IQR 180–240). Participants were asked to keep their eyes open in 12 and closed in 20 of the reviewed studies; 12 articles provided no information. Description of functional MRI acquisition parameters was incomplete in 26 of the 44 analysed studies (61%); methods were judged as not-repeatable in these cases (AXIS item 11). Hemodynamic lags were not considered.

Pre-processing steps common to most studies included slice-time correction; realignment to a reference volume to correct for head motion; normalisation to a template space (usually MNI EPI [64]) including resampling; temporal band-pass filtering (lower end 0.005–0.01 Hz; upper end 0.08–0.15 Hz); and smoothing with a Gaussian filter of full-width at half maximum (FWHM) between 4 and 8 mm.

Confound regression and motion scrubbing were performed and reported less uniformly, as detailed in Tables 1 and 2. Specifically, global signal regression (GSR), that is orthogonalisation of voxel-wise timeseries with respect to the average BOLD signals from the white and grey matter, or the whole brain, was undertaken in 27/44 studies. Twenty-five studies employed subject-wise censoring in which participants were excluded from further analysis if the maximum or average head translation or rotation during the scan exceeded a certain threshold ranging from 0.5 to 3 mm translation and 0.5 to 3° rotation. Ten studies performed volume censoring according to a framewise displacement (FD) or framewise translation/rotation cut-off, excluding participants with too few remaining uncontaminated volumes [31, 49]. Two studies used spike regression [39, 65].

### Connectivity analysis

The majority of studies investigated large-scale functional connectivity between remote brain areas, choosing full or partial temporal correlations between the BOLD time courses as a measure of connectivity. Regions of interest were defined a priori using external brain parcellations in 16 cases. Twenty-seven studies used a data-driven approach such as independent component analysis (ICA), seed-based connectivity analysis (SCA), or local BOLD activity (amplitudes of low-frequency fluctuations [ALFF]) to define regions of interest for further analysis. Many authors interpreted alterations in functional connectivity in the context of a small number of large-scale resting-state brain networks (RSNs), in particular the default mode (DMN) and frontoparietal control (FPCN) networks, but also the dorsal attention (DAN) and salience (SN) networks [22]. Eight reports used graph theoretical approaches, including global network parameters such as efficiency and clustering coefficient [31, 42, 43, 52, 53]; analysis of modularity structures [45, 50]; and self-referential quantification of region-specific centrality [35] to summarise the patterns of connectivity between multiple regions and to thus reflect global organisational principles of the brain networks.

Three studies investigated short-range connectivity [38, 66, 67], using regional homogeneity (ReHo) to quantify the similarity between BOLD signals as a marker of local connectivity [68].

The main findings of individual studies with respect to alterations in resting-state functional connectivity in the context of cerebral small vessel disease are summarised in Table 1. Patterns of altered connectivity were expressed either in comparison to healthy controls or along a gradient of increasing severity of CSVD imaging markers. For clinically or radiologically manifest CSVD, reduced functional connectivity dominated the findings on a global scale [43, 45, 53]. Within resting-state networks, lower functional coupling was repeatedly reported between components of the default mode network [28, 35, 44, 46, 47, 51], which is further supported by the co-occurrence of reduced DMN connectivity and increased WMH burden in patients with non-vascular cognitive impairment [62]. Within the FPCN, reduced connectivity was found in the left insula [46], whereas the right inferior parietal cortex appeared to be more strongly coupled to the rest of this network [51]. The average coupling between the DMN and FPCN was found to be reduced in patients with CSVD [51], even though a small number of inter-network edges showed increased connectivity [39]. The connectivity of the DAN was altered in relation to other networks with increased coupling to the FPCN and reduced coupling to the posterior DMN [39, 51]. The same pattern of altered inter-network connectivity was reported for the salience network [48]; intrinsic connectivity in the SN was increased in patients with CSVD and in association with the extent of white matter disease [83]. In healthy individuals or patients without symptomatic CSVD (Table 2), most studies did not report significant associations between FC and WMH burden [66, 79, 82, 86, 92]. Two studies found an association between higher FC, especially in occipital and frontal areas, and WMH burden [87, 93], whereas in patients with late-life depression the pattern was more similar to the one seen in patients with CSVD [69, 71].

**Table 2:** Summary of included articles analysing resting-state functional connectivity in healthy participants or patients without vascular cognitive impairment. We report imaging and clinical characteristics of patients included in each study, key steps in the acquisition and pre-processing of BOLD data, analysis of functional connectivity, and FC patterns found to be associated with CSVD. Descriptive statistics as extracted from articles are reported as range (min–max) and/or mean ± standard deviation. Missing information is indicted by empty brackets ([]). Reported are clinical characteristics of patients included in each study, details about the quantification of white matter hyperintensities, key steps in the analysis of functional connectivity, and FC patterns found to be associated with CSVD. Arrows indicate increased (↑) or reduced (↓) values, as well as positive (↗) and negative (↘) associations

Reference	Participants	Quantification of WMH load	rs-fMRI acquisition parameters	BOLD pre-processing	FC analysis	FC patterns associated with CSVD
[69]	12 depression 12 HC	Fuzzy connected algorithm [70]	GE Signa, 1.5 T TR 2000 ms, TE 35 ms 64 × 64 × 26, 3.75 × 3.75 × 3.8 mm^3^ [volumes], eyes open	AFNI [Confound regression] [Motion scrubbing]	PCC from ext. template SCA to define DMN Pearson correlation	FC in DMN ↘ WMH in medial PFC
[71]	47 depression 46 HC	Not reported	Siemens Trio, 3 T TR 2000 ms, TE 32 ms 128 × 128 × 28, 2 × 2 × 2 mm^3^ 150 volumes, eyes open	SPM 5 [Confound regression] [Motion scrubbing]	PCC from ext. template SCA to define DMN Pearson correlation	↓ Association between DMN-FC and treatment response after controlling for WMH load
[62]	13 early AD 17 MCI 14 HC	Semi-automatic using FireVoxel [72]	[Scanner] TR 3000 ms, TE 30 ms [matrix], 3.3 × 3.3 × 3.3 mm^3^ 140 volumes, [eyes]	[Confound regression] [Motion scrubbing]	Medial PFC and PCC from ext. atlas SCA to define DMN fALFF	Both increased WMH load and reduced DMN-FC in AD and MCI compared to HC
[73]	100 MCI	In-house automatic pipeline [74]	Philips Achieva, 3 T TR 2000 ms, TE 30 ms [matrix], [resolution] 200 volumes eyes closed	DPARSF Confound regression - 6 motion parameters - GSR: CSF, WM, global [Motion scrubbing]	SCA from the hippocampus and PCC	No association between WMH load and FC
[63]	43 MCI 24 HC	Histogram segmentation [75]	*Training* Philips, 3 T, 8-channel head coil TR 3000 ms, [TE] [matrix], 3.3 × 3.3 × 3.3 mm^3^ 140 volumes eyes open *Testing* Siemens Verio, 3 T, 32-channel head coil TR 2580 ms, [TE] [matrix], 3.5 × 3.5 × 3.5 mm^3^ 180 volumes eyes closed	DARTEL Confound regression - 6 motion parameters - GSR: CSF, WM [Motion scrubbing]	Whole-brain SCA Pearson correlation	No association between WMH and FC
[65]	90 MCI 140 HC	SPM Lesion Segmentation Tool [76]	Siemens Trio, 3 T TR 2300 ms, TE 30 ms [] × [] × 34, 3 × 3 × 4 mm^3^ [volumes] eyes closed	CONN, SPM 12 Confound regression - compCor - GSR: CSF, WM Motion scrubbing - Artefact Detection Tools - Spike regression (FD > 0.5 mm)	Preselected cognitive control networks (FPCN, SN) + DMN Pearson correlation Structural equation modelling	Weaker negative association between executive function/memory and WMH load in patients with high global FC
[77]	18 svMCI + depression 17 svMCI − depression 23 HC	Fazekas scale	GE MR750, 3 T TR 2000 ms, TE 35 ms 64 × 64 × 26, 4 × 4 × 4 mm^3^ 240 volumes [eyes]	DPABI Confound regression - Linear and quadratic trends, 24p - GSR: CSF, WM, global Motion scrubbing - Head motion > 3 mm/3° - Volume censoring (FD > 0.5 mm)	VBM SCA from altered regions Pearson correlation	↑ FC between right middle cingulate cortex and right parahippocampal gyrus
[78]	38 P w Sjogren syndrome 40 HC	Wahlund score	Siemens Trio, 3 T TR 2500 ms, TE 30 ms 96 × 96 × 40, 2.3 × 2.3 × 3 mm^3^ 204 volumes eyes closed	Matlab, DPABI Confound regression - Linear trend - GSR: CSF, WM, global Motion scrubbing - Mean FD > 0.2 mm	SCA from hippocampi Pearson correlation	FC ↗ WMH left hippocampus and right inf. orbital and inf. temporal gyrus
Healthy participants						
[79]	76 healthy participants	Mixture model [80]	GE Signa, 1.5 T TR 2000 ms, TE 40 ms [] × [] × 24, [] × [] × 5 mm^3^ 240 volumes [eyes]	REST Confound regression - Head motion parameters - GSR: CSF,WM, global Motion scrubbing - > 58 outlier volumes (> 1.5 mm/1.5°)	PCC from ext. template SCA to define DMN Pearson correlation	No association between WMH load and FC Episodic memory ↗ medial PFC–left inferior parietal cortex FC in patients with low grey matter volume
[81]	127 healthy (Harvard Ageing Brain Study)	Fazekas 0–1 vs. 2–3	Siemens Trio, 3 T, 12-channel head coil TR 3000 ms, TE 30 ms 72 × 72 × [], 3 × 3 × 3 mm^3^ 124 volumes eyes open	SPM 8 Confound regression - Realignment params + derivatives - GSR: WM, CSF, global Motion scrubbing - Mean FD > 0.15 mm - ≥ 20 outlier volumes (> 0.75 mm/1.5°)	PCC and medial PFC from external DMN template Partial Pearson correlation Probabilistic tractography	↓ Association between PCC-medial PFC FC and mean diffusivity in cingulum bundle
[82]	186 clinically healthy (Harvard Ageing Brain Study)	Automated fuzzy-connected algorithm [70]	Simens Trio, 3 T, 12-channel head coil TR 3000 ms, TE 30 ms 72 × 72 × 47, 3 × 3 × 3 mm^3^ 2 × 124 volumes eyes open	SPM 8 Confound regression - 12 motion parameters Motion scrubbing - ‘mean movement’ > 0.2 mm - > 20 outlier volumes (> 0.75 mm/1.5°)	Template-based rotation to define DMN and FPCN Pearson correlation	No association between WMH load and FC
[83]	51 healthy participants	SPM Lesion Segmentation Tool [76]	Phillips Ingenia, 3 T TR 2600 ms, TE 35 ms 128 × 128 × 35, 1.8 × 1.8 × 4 mm^3^ 125 volumes, [eyes]	REST, GIFT [Confound regression] [Motion scrubbing]	ICA to define DMN, SN, FPN, VN	FC in DMN ↗ WMH in the mediotemporal complex FC in SN ↗ WMH in the right S1 and sup./inf. parietal cortex
[84]	1584 healthy participants (Rotterdam Study)	Tract-specific WMH load [85]	GE Signa, 1.5 T TR 2900 ms, TE 60 ms 64 × 64 × 31, 3.3 × 3.3 × 3.3 mm^3^ 160 volumes, eyes open	FSL Confound regression - Low-frequency drifts - Motion components - ICA Motion scrubbing - Max FD > 0.5 mm, abs. motion > 3 mm	Desikan–Killiany parcellation Pearson correlation Probabilistic tractography	FC ↘ WMH both tract-specific and global
[86]	145 healthy participants	SPM Lesion Segmentation Tool [76]	GE MR750, 3 T TR 1500 ms, TE 27 ms 64 × 64 × 29, 3.75 × 3.75 × 4 mm^3^ 162 volumes eyes open	FSL Confound regression - GSR: CSF, WM Motion scrubbing - FD > 0.5 mm	ICA to define DMN, SMN, FPCN Pearson correlation	No association between WMH load and FC
[87]	69 healthy participants	Coarse-to-fine in-house developed mathematical morphology method [88]	Phillips Achieva, 3 T TR 2050 ms, TE 25 ms 64 × 64 × 47, 3.2 × 3.2 × 3.2 mm^3^ 210 volumes eyes open	CONN Confound regression - 6 motion parameters - GSR: WM, CSF [Motion scrubbing]	AAL atlas, DTI atlas Whole-brain SCA Intrinsic connectivity contrast	FC in the left cuneus and right sup. occipital cortex ↗ WMH in the right ant. corona radiata FC in the left superior occipital cortex ↗ WMH in the right superior corona radiata
[67]	400 healthy participants (Baltimore Longitudinal Study of Aging)	Multimodal supervised classification algorithm [89]	Philips Achieva, 3 T TR 2000 ms, TE 30 ms [matrix], 3 × 3 × 3 mm^3^ 180 volumes [eyes]	Confound regression - 24 motion parameters - GSR: global, WM, CSF Motion scrubbing - ‘summary motion value’ > 0.2 mm - Volume censoring (FD > 0.5 mm, < 5 min)	Geodesic graph-based segmentation Regional homogeneity Sparse connectivity patterns	Pattern of advanced brain ageing characterised by both increased WMH burden and reduced FC compared to resilient agers
[66]	11 healthy participants	Automated regression algorithm [90] using a Hidden Markov Random Field with Expectation Maximization [91]	Siemens Trio, 3 T TR 2000 ms, TE 27 ms 92 × 92 × 43, 2.5 × 2.5 × 3 mm^3^ 240 volumes eyes closed	SPM12 Confound regression - Linear/quadratic, 18 motion parameters - GSR: CSF, WM Motion scrubbing -> 3 mm max, > 3° max -> 24 spikes (FD > 1 mm)	Brainnetome atlas (228) Graph theory to define DMN Pearson correlation	No association between WMH load and DMN FC trajectories
[92]	562 healthy participants	SPM Lesion Segmentation Tool [76]	Phillips Achieva, 3 T TR 2000 ms, TE 20 ms 112 × 112 × 37, 2 × 2 × 3 mm^3^ [volumes], [eyes]	[Confound regression] [Motion scrubbing] Mean FD as covariate in analysis	Desikan–Killiany parcellation FC measure not specified	No association between WMH load and FC
[93]	182 participants (UK Biobank)	BIANCA with manual correction [94]	Siemens Skyra, 3 T TR 735 ms, TE 39 ms 88 × 88 × 64, 2.4 × 2.4 × 2.4 mm^3^ 490 volumes, [eyes]	FMRIB (FSL), ICA-FIX Confound regression - ICA [Motion scrubbing]	ICA, AAL atlas Pearson correlation Degree centrality	FC ↗ WMH in right orbitofrontal cortex
[95]	250 healthy (Harvard Aging Brain Study)	Automated fuzzy-connected algorithm [70]	Siemens Trio, 3 T TR 3000 ms, TE 30 ms [matrix], 3 × 3 × 3 mm^3^ 2 × 124 volumes, eyes open	SPM 8 Template-based rotation method [Confound regression] [Motion scrubbing]	Template-based rotation to define DMN, SMN, DMN, and FPCN Pearson correlation	Association between WMH load and FC not investigated FC in DMN ↘ risk of progression to MCI

### Assessment of cognitive impairment

In the majority of studies, cognitive testing on participants was performed and investigated in association with the extent of white matter disease and functional connectivity. In addition to scales covering the global level of cognitive functions and deficits (MMSE, MoCA, and CDR), impairments in specific cognitive domains were quantified by sub-scores of these global scales or specialised neuropsychological test batteries, operationalising, in particular, executive function, processing speed, and memory. Table 3 summarises key findings of individual studies in these different domains. Most studies were able to confirm known associations between CSVD and cognitive impairment on the one hand, and, albeit less robustly, between functional connectivity and cognitive impairment on the other hand. Only few articles, however, addressed the question of how structural white matter damage and functional connectivity interact to affect cognition. In one analysis of 127 clinically healthy participants of the Harvard Ageing Brain Study, it was shown that the extent of WMH-associated decoupling of structural and functional connectivity in the default mode network correlated with both executive function and memory [81]. Moreover, in a combined analysis of 140 healthy participants and 90 patients with both vascular and non-vascular cognitive impairment, the authors demonstrated that the association of higher WMH load with poorer executive function and memory scores was moderated by global functional connectivity in the FPCN and by local FC in the salience network [65].

**Table 3:** Summary of reported associations between altered FC patterns in CSVD and cognitive ability. Arrows indicate positive (↗) and negative (↘) associations

Cognitive domain	Reference	Instruments	
General	[47]	–	MMSE ↗ FC between the left thalamus–left orbitofrontal lobe
	[38]	–	MMSE ↗ ReHo in the right angular gyrus and precuneus MoCA ↗ ReHo in the bilateral angular gyrus, the right precuneus, medial/dorsolateral PFC, and supplementary motor area
	[39]	–	MMSE ↗ FC right precentral–right calcarine fissure, left posterior inferior parietal lobe–left Heschl, right posterior inferior parietal lobe–right dorsolateral PFC MMSE ↘ FC right posterior inferior parietal lobe–left Heschl, left intraparietal sulcus–right superior temporal gyrus MoCA ↗ FC right posterior inferior parietal lobe–right anterior PFC, right posterior inferior parietal lobe–right dorsolateral PFC MoCA ↘ FC right posterior inferior parietal lobe–left Heschl, left intraparietal sulcus–right superior temporal gyrus
	[53]	–	MoCA ↗ small-worldness
	[33]	–	MMSE ↘ parieto-occipital FC in patients with confluent WMH
Executive function	[35]	CERAD battery [96]	Phonemic fluency ↗ FC in bilateral sup. parietal lobe, SMA, premotor cortex, MCC, and posterior superior frontal sulcus RT_Stroop, neutral_ ↘ FC in the bilateral premotor cortex, superior frontal sulcus, left inferior frontal sulcus, left SMA, left middle temporal sulcus, and right MCC RT_Stroop, neutral_ ↗ FC in the inferior parietal lobe and cerebellar lobules Crus II, VIIb, and VIII RT_TMT-A_ ↘ FC in the bilateral premotor cortex, left posterior middle frontal gyrus, left inferior frontal sulcus, right superior parietal lobe, and left SMA RT_Stroop, incongruent_ ↘ FC in the left premotor cortex/posterior middle frontal gyrus RT_Stroop, incongruent_ ↗ FC in the cerebellar regions VI, Crus I, and Crus II
	[81]	Letter/category fluency, letter-number sequencing of the WAIS-III, Digit Span Backward of the WAIS revised (WAIS-R), Self-Ordered Pointing task, mod. Flanker task, and TMT A/B Confirmatory factor analysis [97]	Executive function ↗ FC-SC decoupling in DMN
	[36]	Visuospatial/executive sub-score of MoCA	Executive function ↗ FC in the right cingulate motor area
	[83]	Stroop test	Time interference index ↗ FC in anterior DMN and SN
	[65]	TMT A/B, Stroop test Latent variables	Association (executive function ↘ WMH) attenuated in patients with high global FC in FPCN Associations (executive function ↘ WMH) and (memory ↘ WMH attenuated) in patients with high local FC in SN
	[51]	TMT-A/B, Stroop test	RT_TMT-A_ ↘ FC in FPCN in the right inferior parietal lobule RT_TMT-A_ ↘ FC between the dorsolateral PFC and DMN between bilateral PCC and right precuneus
	[43]	Semantic similarity test Stroop test	Mean FC ↗ similarity index Stroop C score ↗ path length, ↘ global efficiency
Memory	[45]	Auditory Verbal Learning Test [AVLT] [98]	Delayed recall ↘ participation coefficient left superior parietal lobule Recognition ↘ characteristic path length
	[65]	AVLT, structural equation modelling	Memory ↗ WML*global FC
	[52]	AVLT	FC ↘ long recall between right olfactory–right rectus; ↘ short recall between right olfactory–left pallidum FC ↗ RT_TMT-A_ between right olfactory–left pallidum

### Risk of bias and confounding

**Fig. 2:**
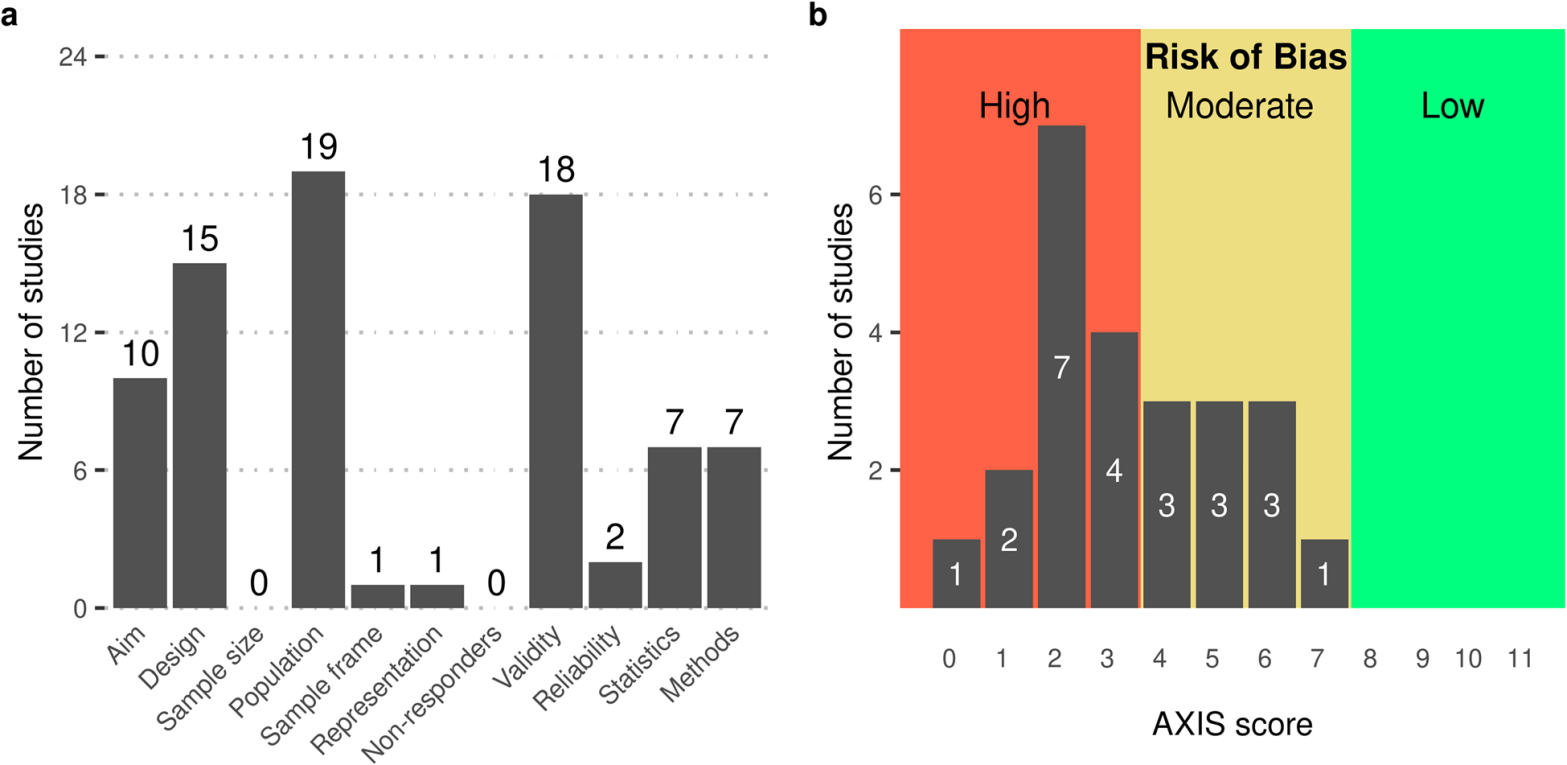
Assessment of risk of bias of 24 reviewed articles using the Appraisal tool for Cross-Sectional Studies (AXIS). **a** Distribution of per-item scores, indicating, for each item, the number of articles satisfying its definition. A detailed description of AXIS items and shortcomings of individual articles is presented in Additional file 1: Tables S1 and S2. **b** Distribution of aggregate AXIS scores computed as the number of items satisfied by any given reviewed article. Trichotomisation of the theoretical range 0–11 leads to the risk of bias being judged as high (0–3), moderate (4–7), or low (8–11)

Risk of bias was assessed using the AXIS tool for all 24 studies recruiting patients with clinical CSVD. We did not formally assess the risk of bias in studies reporting results on FC and WMH in the context of conditions different from vascular cognitive impairment or in longitudinal studies. According to the AXIS tool, all studies thus assessed had an at least moderate risk of bias (10/24 moderate, 14/24 high). The distribution of assessments of individual quality items of the tool is depicted in Fig. 2 a. The overall aim or objective of the study (Item 1) was deemed unclear in 14 studies, often because of a lack of distinctions between exploratory and confirmatory, and causal and correlational approaches. In 9 cases, where aims included the inference of causal effects or were too broad to be assessed, a cross-sectional design was judged as inappropriate (Item 2). The sample size was not satisfactorily justified in any study. The reference population (Item 4) was mostly adequately specified as patients with CSVD, qualified by lists of inclusion and exclusion criteria. In five studies, the definition of the target population was unclear or contradictory. All but one article reported results from single-centre studies that recruited a convenience sample from a clinical setting; in these cases, the sample frame (Item 5) was judged as inappropriate and the selection process (Item 6) as non-representative. The exceptions were an analysis of a formal clinical register [84, 99]. No article addressed non-responders. Risk factors and outcomes (Item 7) were mostly valid (see above); exceptions included one unvalidated method to quantify WMH load [87] and the use of global graph parameters such as efficiency and path length. Reliability of outcome measures (Item 9) was generally judged to be low given the poor reproducibility of FC estimates in the context of CSVD, except for studies who explicitly estimated reliability as part of the study design [31, 42]. There were two main problems with the statistical methods used: firstly, confusion of exploratory and confirmatory approaches (cf. Item 1) led to a lack of clearly specified hypotheses and thus to inappropriately controlled type-I error rates in the case of multiple testing; secondly, many papers employed multi-scale approaches, in which results from the first, often global, analyses informed hypotheses tested in later, often more local, analyses. It is known that this method can inflate the rate of false-positive findings if the entire analysis pipeline is not accounted for properly, for example in a bootstrap loop [100]. The quality of the description of methods varied considerably. No article provided links to the program code used in the analysis, but this was not required to satisfy Item 12. Specific shortcoming included incomplete reporting of MRI acquisition parameters, lack of description of structural image pre-processing, and lack of detail in the description of statistical methods, such as choice of covariates, method to determine of *p*-values, or correction for multiple testing. The distribution of aggregate AXIS scores is shown in Fig. 2b. Given that none of the included studies had a low risk of bias or was preregistered, the overall risk of bias in the reviewed literature seems high.

Cardiovascular risk factors such as age, hypertension, diabetes mellitus, and dyslipidaemia are known to be associated with imaging markers of CSVD [101]. They also affect cerebrovascular reactivity and the circulatory autoregulation in response to neuronal activity (*neurovascular coupling*) [29] and are thus potential confounders of the relation between WMH and BOLD-derived functional connectivity. Similarly, vasoactive medications, in particular antihypertensives, which are commonly prescribed to patients with CSVD as well as substances like nicotine or caffeine may alter neurovascular coupling [102]. Despite this, reporting of and adjustment for comorbidities and medication was poor in the reviewed studies. While information on the demographic variables age and sex was provided in all reviewed articles, only about half reported results of analyses adjusted for these factors. Nine articles gave details on cardiovascular risk factors, yet none attempted to control for their potential confounding effect. Effects of prescribed medication or caffeine intake were not considered.

## Discussion

For this systematic review, we identified 44 articles published in the previous 10 years reporting on MRI-derived resting-state functional brain connectivity in patients with white matter hyperintensities of presumed vascular origin as a marker of cerebral small vessel disease. Based on patient characteristics and research objective, studies could be divided into three groups: (1) group comparisons of patients with clinically and/or radiologically manifest CSVD, often involving a control group of healthy participants or patients with CSVD at different levels of cognitive impairment; (2) cohort studies of clinically healthy individuals in which white matter hyperintensities are reported as one of several parameters, often with the aim of characterising structure–function relationships or patterns of brain ageing; (3) investigations of resting-state connectivity in clinical conditions not primarily related to vascular pathology, in which measures of white matter disease were reported as covariates.

The overall median sample size of included studies was 68. There was a stark contrast in sample size between studies of patients with symptomatic CSVD (median 58, IQR 46–84, *n* = 24) and studies of clinically healthy participants (median 145, IQR 73–293, *n* = 12). Samples in studies focusing on non-vascular clinical conditions were of intermediate size (median 73, IQR 55–95, *n* = 8). These differences might be due to increased complexities associated with recruiting patients in a clinical context or the fact that some of the larger studies used data from comprehensive population-based research efforts, such as the Rotterdam Study [84, 103], the Harvard Brain Ageing Study [81, 82, 104], or the UK Biobank [93, 105].

### Operationalisation of CSVD is study-context dependent

In addition to sample size, groups of studies also differed in their approaches to quantifying the severity of white matter disease. Clinically focused studies tended to rely on validated rating scales, such as the Fazekas or Wahlund scale, which assign an ordinal score based on the extent and distribution of white matter hyperintensities on T2-weighted MR imaging. A minority of studies considered the presence of lacunar infarcts as an additional marker for CSVD. The population-based studies of healthy participants, on the other hand, employed the cumulative volume of WMH as a continuous measure of disease burden. Numerical lesion load has the advantage of providing better resolution of inter-individual differences in groups of mildly affected participants; in addition, it can be determined reasonably reliably using automatic or semi-automatic image processing methods, although some degree of manual post-processing was usually done in the studies reviewed here [106]. Brain atrophy as a structural marker of both CSVD and neurodegenerative disease is known to be associated with changes in intrinsic brain connectivity [107]; it was included in many of the population-based studies using either the total intracranial volume to normalise observed WMH loads or region-specific grey matter volume, such as can be obtained from voxel-based morphometry (VBM) or cortical thickness measurements. Although methods have been developed to segment perivascular spaces (PVS) and cerebral microbleeds in an automated fashion [108–114], none of the reviewed articles utilised enlarged PVS, and only one used microbleeds [49] as a marker of CSVD.

The variety of qualitative and quantitative analysis methods reflects the clinical heterogeneity of study populations comprising patients with CSVD at different stages of the disease. An attempt at standardising the assessment and reporting of imaging markers of CSVD was made in the STandards for ReportIng Vascular changes on nEuroimaging (STRIVE) position paper [6]. However, despite being published in 2013, the definitions and recommendations outlined in the STRIVE were referenced in only six of the 33 reviewed papers published after 2013 [31, 50–52, 62, 108].

### Functional connectivity methods reflect clinical heterogeneity

The analysis of recorded BOLD signals has not been standardised, with a broad variety of coupling measures and dimensionality reduction techniques being at the disposal of the researcher [20]. All reviewed studies used Pearson’s correlation coefficient to quantify the synchrony between BOLD time series in different parts of the brain. No clear distinction between full and partial correlations was often made, thus making the interpretation of direct or indirect connectivities difficult [115]. Similarly, the handling and interpretation of negative correlations was rarely reported or discussed [116–118]. None of the included articles attempted to estimate directed [119–121] or time-varying functional connectivities [122–124], or to quantify patterns of synchronous activity involving more than two regions [125, 126]. Analytical approaches included whole-brain analyses (eigenvector centrality, connectivity density); the investigation of functional connectivities between region of interests, often components of well-defined intrinsic resting-state networks, which were either derived from the data themselves (independent component analysis) or specified a priori by an external brain parcellation; and combinations of the two (seed-based correlation analysis).

Brain parcellations for the region-of-interest-based analyses were mostly based on anatomically defined atlases, such as the automatic anatomical labelling (AAL) atlas [127], the Desikan–Killiany parcellation [128], or the H-1024 random parcellation [129], which do not take into account the functional architecture of the brain. Only three very recent articles [31, 49, 66] used the multimodal brain parcellations of Power [130] or Schaefer [131], or the Brainnetome atlas [132], which have been shown to better respect the functional organisation of the brain [22]. In addition to interpreting changes in functional connectivity directly, a few studies attempted to summarise patterns of FC by measures of global network organisation using graph theory. These approaches have been instrumental in the study of complex brain networks and include parameters to reflect the notions of integration, such as efficiency or characteristic path length; segregation, such as clustering coefficients; or community structure, quantified by modularity scores and participation coefficients [9, 133, 134]. With the exception of community detection, however, these network parameters have been defined only for structural brain networks and lack validation for networks derived from functional connectivity [135].

### Structure–function coupling shapes the impact of CSVD

The large-scale temporospatial organisation of neuronal activity in the brain is known to be supported and constrained by the anatomy of axonal projections that form structural connections between both adjacent and remote brain areas [136, 137]. This coupling between structure and function is particularly pronounced in the default mode network [138], possibly reflecting the long periods of time that the brain is engaged in inward-directed thought, memory formation and retrieval, or social estimation [139]. While the structural connectome thus contributes to maintaining stable neural activity patterns, it also means that normal functional connectivity is vulnerable to damage to white matter pathways as occurring in CSVD [140]. Most articles included in this review quantified the extent of white matter damage by using either neuroradiological rating scales or total lesion volume, as detailed above. Such global approaches are, however, not able to differentiate between lesions in functionally silent brain areas that can more easily be compensated by rerouting information through alternative redundant pathways, and lesions in functionally critical, strategic locations, where even spatially limited damage can be associated with substantial behavioural sequelae. In the context of cognition, damage to subcortical nuclei and tracts with a high density of neuromodulatory projections such as the dorsomedial and anterior thalamic nuclei or the anterior limb of the internal capsule appears to be particularly consequential [24, 52, 87, 141]. Advanced diffusion-weighted structural imaging modalities allow the spatial mapping of fibre tracts and the quantification of tract-specific white matter lesion loads [142, 143]. In combination with resting-state BOLD imaging, this approach has been used to show that leukoaraiosis disrupts functional connectivity in a spatio-topological non-uniform way that is shaped by the anatomy of the brain’s white matter scaffold [84]. The strongest association between tract-specific ischaemic damage and reduced FC was observed in the fronto-occipital fasciculus, which supports connectivity between the salience and frontoparietal control networks [144]. In addition to affecting functional connectivity directly, ischaemic white matter disease also seems to exert an indirect effect by modulating the coupling between structural and functional connectivity. Specifically, the association between mean diffusivity in the cingulum bundle and functional connectivity between the medial prefrontal and posterior cingulate cortices was significantly attenuated in patients with higher WMH burden, thus contributing to decoupling the anterior and posterior parts of the default mode network [81].

Both structural and functional connectomes share properties of complex networks, such as the presence of network communities, high-clustering with short path length (small-worldness), and hierarchical organisation [11, 145]. With cognition considered an emergent property of distributed neuronal activity in the brain [146, 147], understanding the behavioural sequelae of CSVD requires an understanding of how ischaemic lesions disturb not only specific fibre tracts and functional connections but also the global organisation of synchronous activity. Graph theoretical analyses have suggested that the global topology of functional brain networks in the presence of CSVD exhibits increased path length and modularity and reduced small-worldness that correlated with cognition [45, 53]. A similar effect was also observed in the structural networks of patients with CSVD and ischaemic stroke [13, 15, 148–150].

An intriguing open question is the differentiation between altered functional connectivity as a direct consequence of damage to the supporting fibre tracts, and compensatory changes. The latter are thought to contribute to maintaining normal cognitive function in the early stages of the disease [83]. Indeed, increased coupling between brain areas has repeatedly been reported in cognitively normal individual with white matter hyperintensities [78, 87, 93].

### Resting-state FC informs an updated disconnection hypothesis

The association of white matter hyperintensities of presumed vascular origin with cognition has been extensively described [151–153], and indeed, cognitive impairment is one of the clinical hallmarks of manifest cerebral small vessel disease [154]. On the other hand, resting-state fMRI connectivity has been found useful in extracting neural correlates of cognitive function and mood disorders [155, 156]. Under normal physiological circumstances, patterns of coordinated activity within and between a small number of large-scale intrinsic brain networks have emerged as particularly relevant [146], including activation of the default mode network in brain states characterised by self-referential thought or rest that is anti-correlated with activation of the dorsal attention network; deactivation of the default mode network during focused attention on external stimuli [157]; and a modulating role of a frontoparietal control network with increased connectivity to the DMN as a correlate of working memory performance [158, 159]. Building upon these ‘cornerstones’ of functional connectivity under normal physiological circumstances, a disconnection hypothesis has been developed that postulates reduced DMN and FPCN connectivity, decoupling of neuronal activity along the anterior–posterior axis, and functional disconnection of the prefrontal cortex as neuronal correlates of cognitive impairment in CSVD [24]. This model is supported by several recent studies that reported decreased functional connectivity between the medial PFC and posterior components of the DMN in patients with CSVD [28, 44, 47], and observed an association with reaction times in the Stroop test [35]. A behaviorally relevant dissociation in functional resting-state fMRI activity and local connectivity was found between the anterior and posterior parts of the DMN with lower ReHo and ALFF values in the medial PFC and higher values in the precuneus and posterior cingulate cortex in patients with CSVD compared to healthy controls [38, 44]. Both increases and decreases of FC within the FPCN and DAN as well as their coupling with the DMN have been reported to be associated with CSVD [39, 46, 51], but the heterogeneity of these results and limited correlation with cognitive test scores makes it difficult to distinguish primary effects of disconnection from compensatory changes or sampling variability without physiological relevance.

In addition to these established networks, connectivity patterns of the salience network (SN) have recently been investigated, with increased SN-FPCN and SN-DMN couplings associated with small vessel disease [51]; additionally, increased connectivity within the SN in patients with CSVD was associated with worse performance in the Stroop interference test [83]. In patients with mild cognitive impairment, the association between white matter disease and executive function was attenuated in the presence of increased local connectivity of the salience network [65]. The salience network includes the anterior insula, the dorsal anterior cingulate cortex, and subcortical components. Similar to the FPCN, it has a critical role in switching activity between different brain networks and has been implicated as a key component in network models of neuropsychiatric disorders [160–162]. Specifically, increased connectivity within the SN and altered SN-DMN and SN-FPCN coupling have been described in patients with Alzheimer’s disease and mild cognitive impairment [163–165].

Community-dwelling adults with early CSVD often perform normally on neuropsychological tests and only report mild subjective cognitive deficits [166]. This preclinical stage has been linked to compensatory mechanisms especially in patients who benefit from a larger cognitive reserve [167, 168]. Three recent studies provide further evidence for this hypothesis, linking increased functional connectivity to frontal and temporal areas to ischaemic white matter lesion load in cognitively normal subjects [78, 83, 93].

### Current knowledge is limited by the risk of bias, confounding, and methodological constraints

While it is possible to extract consistent themes from the reviewed articles that point toward physiologically relevant patterns of altered FC in the context of CSVD and cognitive impairment, the current literature is characterised by a high degree of heterogeneity of individual results. As discussed above, this may partly reflect variability in pre-processing and analytical approaches as well as heterogeneity in the clinical populations under investigation. However, given the absence of preregistered reports or high-quality multi-centre studies and the predominantly moderate-to-high risk of bias in individual studies, it must be assumed that selective reporting allowed the literature to be contaminated by a substantial number of false-positive findings, reflecting spurious associations and group differences. In addition, it is possible that reported results are confounded by the presence of other age-related pathology or neurodegenerative comorbidities, such as Alzheimer’s disease [169], which were considered specifically in only a small minority of studies.

Comparison and synthesis of individual study findings is further hampered by differences in data cleaning techniques, which are known to influence functional connectivity estimates [170]. Two important dimensions of BOLD pre-processing relate to removal of the global signal from the whole brain or tissue type compartments, and handling of subjects or frames with high motion. Global signal regression is known to be effective at mitigating the widespread inflation of connectivity estimates induced by subject motion, resulting in an elevated distance-dependence of residual motion artefacts [171]. Despite this theoretical prediction and the observation that GSR might improve associations between FC and behavioural measures [172], the use of GSR was not associated with specific patterns of altered connectivity or stronger relations with cognitive measures in the reviewed papers. Similarly, no clear effect of different motion scrubbing strategies, i.e. the censoring of subjects or individual volumes due to excessive average or framewise displacement, could be recognised. It seems likely that the myriad of unstandardised pre-processing choices is contributing to the heterogeneity of published results and that findings which have not been shown to be robust with respect to such choices should therefore be interpreted with great care.

Even ignoring potential biases inherent in study design and publication practice, the study of FC in the context of CSVD may be limited by more fundamental obstructions. One concern is that the reliability of estimating functional connectivity may be negatively affected by the presence of white matter lesions itself. Two of the reviewed studies reported results from repeated measurements on participants in longitudinal designs [31, 42]. Worryingly, in both cases, resting-state fMRI measures were found to be poorly reproducible, indicating a further need to evaluate their robustness as an imaging biomarker. In one case, this might have been a consequence, in part, of using a brain parcellation that does not respect the functional boundaries between brain areas, which is known to be damaging to network estimation [119]. However, the persistence of low reliability measures for a range of network characteristics across network densities and atlas resolutions, as well as the particularly poor reproducibility of functional network measures in patients with CSVD compared to controls, suggests more fundamental problems beyond the choice of parcellation. The finding of poor reproducibility of RSNs and graph metrics in CSVD contrasts with high reproducibility reported in healthy participants [173–176] and patients with stable multiple sclerosis [177–179]. It has been suggested that age and confounding age-related pathologies could be responsible for reduced reliability of functional connectivity estimates [180, 181]; however, specific methodological challenges arise in patients with cerebral small vessel disease as a consequence of microvascular pathology, that are absent in other conditions.

As a measure of synchronous brain activity, the interpretation of BOLD-derived functional connectivity is contingent upon an understanding of the relation between neuronal activity and local blood flow. This neurovascular coupling, however, is known to be altered in normal ageing as well as the presence of ischaemic disease [102, 182, 183], and attributing differences in BOLD-derived measures of connectivity to either vascular or neuronal factors is therefore challenging [184]. More specifically, white matter lesions of presumed vascular origin are known to be associated with subcortical hypoperfusion [185], possibly reflecting observed rarefaction of the microcirculation in a mouse genetic model of CSVD [186]. The later stages of neurovascular coupling involve dynamic upregulation of regional blood flow mediated by increased CO_2_ concentration in areas of increased neuronal activity [102]. This mechanism appears to be affected in the presence of CSVD as demonstrated by a diminished cerebrovascular response to hypercapnia in an early study involving 24 patients with leukoaraiosis [187], and an association between WMH load and sonographically assessed measures of pulsatility and dynamic autoregulation in a cohort of elderly patient with cardiovascular risk factors [188]. These findings are further complicated by differences in age-related changes in cerebrovascular reactivity between grey and white matter [189]. BOLD-derived functional connectivity is a function of BOLD activity in remote brain areas, and spatial variations in age- or disease-related changes in neurovascular coupling might therefore affect FC estimates in unpredictable ways [190]. A small study of 25 subjects with WMH found that while cardiovascular risk factors are associated with cerebrovascular reactivity, no such association was observed for resting-state functional connectivity in the default mode network [29]. One potentially testable hypothesis about the effects of impaired neurovascular coupling on functional connectivity estimates derives from the observation that BOLD-derived measures of synchronous brain activity are a composite of true coincident neuronal activation (‘signal’) and shared noise, where the latter tends to be more dominant for short-range connections [171]. Reduced ‘signal’ strength as a consequence of a lower vascular response would therefore be expected to result in weaker and less precise FC estimates, especially in long-range connections.

### Limitations

While being comprehensive in our inclusion of primary research articles from electronic databases and other sources, we cannot exclude the possibility that additional findings from the grey literature, such as blogs or unpublished conference abstracts, have not been covered by this review. In order to keep the scope of the work focused, we have not included reports of task-based connectivity or resting-state connectivity derived from electrophysiological recordings. Findings obtained using these alternative paradigms and modalities might lend further support to the themes of disturbed connectivity patterns outlined above. This review attempted a qualitative synthesis of the recent literature; the heterogeneity of study designs and populations did not permit the extraction and quantitative analysis of numerical effect estimates beyond sample size and age of participants. From a meta-analytical perspective, it can be noted, however, that all studies of patients with clinically manifest CSVD report significant FC alterations, while that is the case for only 30% of the population-based studies despite larger sample sizes. This discrepancy could reflect larger effect sizes in clinically preselected patients or indicate selective reporting in the sense of publication bias [191].

For conciseness, we have concentrated our attention on cognitive impairment as one of the main clinical sequelae of CSVD. Associations of altered patterns of functional connectivity with depressive symptoms, apathy, or gait imbalance were rarely reported and have not systemically been explored here. As an entry point to the recent literature, we note that abnormal functional coupling has been observed as a correlate of late-life depression in the context of the vascular depression hypothesis [192–195]; while apathy has been investigated using resting-state fMRI in various clinical contexts [196–198], results on gait disorders are scarce [34, 199]. Functional connectivity does not seem to interact with race or socio-economic status as possible contributing factors to neurodegeneration [200].

## Conclusion

The large number of recent studies investigating resting-state fMRI connectivity in the presence of cerebral small vessel disease reflects an active ongoing interest to understand the interplay between structural brain damage, associated changes in the spatiotemporal organisation of neural activity, and clinical sequelae. The literature documents accumulating evidence for a network disruption model underlying cognitive impairment in CSVD that is characterised by disordered connectivity patterns in the DMN and FPCN and a decoupling of neuronal activity along the anterior–posterior axis, mediated by structural damage to long association tracts and cortico-subcortical connections. In addition, evidence is emerging that altered connectivity of the salience network might be a novel neuronal correlate of cognitive deficits in patients with CSVD.

The synthesis of population-based studies involving healthy participants with low white matter disease burden and clinical studies recruiting patients with manifest CSVD suggests a pattern of increased functional connectivity in various frontal and temporal brain areas consistent with compensatory upregulation at low white matter disease burden in the early stages of the disease, and dysfunctional patterns of functional connectivity among distributed brain networks in more severely affected patients, possibly reflecting a break-down of compensatory mechanisms as the disease progresses and cognitive symptoms develop.

Further research is needed to address the problem of poor reproducibility of resting-state functional brain networks in patients with CSVD and to establish interacting effects of white matter damage of presumed vascular origin and functional reorganisation on cognition in preregistered, sufficiently powered, longitudinal studies. We expect particularly useful insights from multimodal investigations that combine resting-state and task functional MRI with electrophysiological recordings or metabolic imaging to improve temporal resolution and infer cellular processes relating to pathology.

## Supplementary Information

**Additional file 1:** Search strategy. Detailed description of search parameters to identify relevant literature. Risk of bias assessment. Supplementary methods and results relating to the assessment of bias in individual studies. **Table S1.** Description of items used to score risk of bias. **Table S2.** Results of risk-of-bias assessments using the AXIS tool.

## Data Availability

All data generated or analysed during this study are included in this published article and its supplementary information files.

## Abbreviations

AAL: Automatic anatomical labelling
ACC: Anterior cingulate cortex
AD: Alzheimer’s disease
AFNI: Analysis of Functional NeuroImages
ALFF: Amplitude of low-frequency fluctuations
AN: Auditory network
ANTs: Advanced Normalisation Tools
AROMA: Automatic Removal of Motion Artifacts
AVLT: Auditory verbal learning test
AXIS: Appraisal tool for Cross-Sectional Studies
BRAMILA: BRAin and MInd LAb
CADASIL: Cerebral autosomal dominant arteriopathy with subcortical infarcts and leukoencephalopathy
CDR: Clinical Dementia Rating scale
CERAD: Consortium to Establish a Registry for Alzheimer’s Disease
CN: Cognitively normal
compCor: Component-based noise correction
CONN: Functional connectivity toolbox
CSF: Cerebrospinal fluid
CSVD: Cerebral small vessel disease
DAN: Dorsal attention network
DARTEL: Diffeomorphic Anatomical Registration using Exponentiated Lie algebra
DMN: Default mode network
DPABI: Data Processing & Analysis for Brain Imaging
DPARSF: Data Processing Assistant for Resting-State fMRI
DTI: Diffusion tensor imaging
EEG: Electroencephalography
fALFF: Fractional ALFF
FC: Functional connectivity
FD: Framewise displacement
FLAIR: Fluid-attenuated inversion recovery
FPCN: Frontoparietal control network
FSL: Functional Magnetic Resonance Imaging of the Brain Software Library
GIFT: Group ICA Of fMRI Toolbox
GRETNA: GRaph thEoreTical Network Analysis
GSR: Global Signal Regression
HC: Healthy control
ICA: Independent component analysis
MCC: Middle cingulate cortex
MCI: Mild cognitive impairment
MMSE: Mini Mental State Exam
MoCA: Montreal Cognitive Assessment
MRI: Magnetic resonance imaging
NBS: Network-based statistics
PET: Positron emission tomography
PCC: Posterior cingulate cortex
PFC: Prefrontal cortex
PiB: Pittsburgh compound B
PRISMA: Preferred Reporting Items for Systematic Reviews and Meta-Analysis
ReHo: Regional homogeneity
REST: REsting State fMRI data analysis Toolkit
ROI: Region of interest
RSN: Resting-state network
RT: Reaction time
SC: Structural connectivity
SCA: Seed-based connectivity analysis
SMA: Supplementary motor area
SMN: Somatomotor network
SN: Salience network
SPM: Statistical Parametric Mapping
STRIVE: STandards for ReportIng Vascular changes on nEuroimaging
SVaD: Subcortical vascular dementia
svMCI: Subcortical vascular MCI
T: Tesla
TE: Echo time
THA LAC: Thalamus lacune
TMT: Trail making test
TR: Repetition time
VBM: Voxel-based morphometry
VN: Visual network
WAIS: Wechsler Adult Intelligence Scale
WM: White matter
WMH: White matter hyperintensity
