# Supplementary material for: Functional connectivity changes in cerebral small vessel disease - a systematic review of the resting-state MRI literature
Source: BMC Med. 2021 May 5;19:103. doi: 10.1186/s12916-021-01962-1 (PMC8097883; doi:10.1186/s12916-021-01962-1)
Supplement: Supplementary file 1 — Additional file 1: Search strategy. Detailed description of search parameters to identify relevant literature. Risk of bias assessment. Supplementary methods and results relating to the assessment of bias in individual studies. Table S1. Description of items used to score risk of bias. Table S2. Results of risk-of-bias assessments using the AXIS tool. [file 12916_2021_1962_MOESM1_ESM.doc]

Supplementary Information (Additional file 1) to

## Functional connectivity changes in cerebral small vessel disease – a systematic review of the resting-state MRI literature

Maximilian Schulz^1^, Caroline Malherbe, PhD^1,2^, Bastian Cheng, MD^1^, Götz Thomalla, MD^1^, Eckhard Schlemm, MBBS, PhD^1,✉^

^1^ Department of Neurology, University Medical Centre Hamburg-Eppendorf, Hamburg, Germany

^2^ Department of Computational Neuroscience, University Medical Centre Hamburg-Eppendorf, Hamburg, Germany

^✉^Address for correspondence

Dr. Dr. Eckhard Schlemm
Universitätsklinikum Hamburg-Eppendorf
Kopf- und Neurozentrum
Klinik und Poliklinik für Neurologie
Martinistr. 52
20251 Hamburg

GERMANY

e.schlemm@uke.de

# Search strategy

The PubMed online database was searched thrice on 1 December 2019 and again on 28 June 2020 and 22 November 2020. The following search string was used

*(SVD OR "small vessel disease" OR "small-vessel disease" OR WML OR WMH OR "white matter lesion*" OR "white matter hyperintens*" OR "white-matter lesion*" OR "white-matter hyperintens*" OR leukoaraiosis OR microangiopath*) AND (connectivity OR network* OR graph OR hub OR modul*) AND (MRI AND (functional OR BOLD OR "resting-state" OR "resting state"))*

The “Best Match” algorithm (Fiorini, Canese, *et al.*, 2018) was selected and search results were limited to articles in English published after 1 January 2010.

# Risk of Bias assessment

Risk of bias in cross-sectional studies focusing on patients with manifest CSVD was assessed using the Appraisal tool for Cross-Sectional Studies (AXIS tool)[26], modified to not contain items related to presentation of Results, the Discussion of findings, or the Funding of the study [27]. The eleven items thus assessed are shown in Table S1.

|  | *Label* | *Description* |
| --- | --- | --- |
| *Item 1* | Aims / objectives | Were the aims/objectives of the study clear? |
| *Item 2* | Study design | Was the study design appropriate for the stated aim(s)? |
| *Item 3* | Sample size | Was the sample size justified? |
| *Item 4* | Target population | Was the target/reference population clearly defined? (Is it clear who the research was about?) |
| *Item 5* | Sample frame | Was the sample frame taken from an appropriate population base so that it closely represented the target/reference population under investigation? |
| *Item 6* | Representative selection | Was the selection process likely to select subjects/participants that were representative of the  target/reference population under investigation? |
| *Item 7* | Addressed non-responders | Were measures undertaken to address and categorize non-responders? |
| *Item 8* | Validity | Were the risk factor and outcome variables measured appropriate to the aims of the study? |
| *Item 9* | Reliability | Were the risk factor and outcome variables measured correctly using instruments/measurements that had been trialed, piloted or published previously? |
| *Item 10* | Statistics | Is it clear what was used to determined statistical significance and/or precision estimates? (e.g. p-values, confidence intervals) |
| *Item 11* | Repeatability | Were the methods (including statistical methods) sufficiently described to enable them to be repeated? |

Table S1 Items of the AXIS tool. Adapted from [26]. *Description of the eleven included scores for each individual article and, where appropriate, explanatory notes, are presented in Table S2.*

|  |  | ***Risk of bias*** | ***INTRODUCTION*** | ***METHODS*** | | | | | | | | | |
| --- | --- | --- | --- | --- | --- | --- | --- | --- | --- | --- | --- | --- | --- |
|  |  |  | **1** | **2** | **3** | **4** | **5** | **6** | **7** | **8** | **9** | **10** | **11** |
|  | Sun, Qin, *et al.*, 2011 [36] | 2 | 🗶 Unclear | 🗶 | 🗶 | ✓ | 🗶 | 🗶 | 🗶 | ✓ | 🗶 | 🗶 not enough details given | MRI acquisition params incomplete |
|  | Yi, Wang, *et al.*, 2012 [59] | 2 | 🗶 Exploratory or confirmatory ?causal hypothesis | 🗶 | 🗶 | ✓ | 🗶 | 🗶 | 🗶 | ✓ | 🗶 | 🗶 Handling of covariates | Structural image pre-processing missing |
|  | Schaefer, Quinque, *et al.*, 2014 [52] | 1 | 🗶Exploratory or confirmatory | ✓ | 🗶 | 🗶 "early SVD" | 🗶 | 🗶 | 🗶 | 🗶 EV centrality | 🗶 | 🗶 t-tests with covariates Multi-stage analysis not accounted for | MRI acquisition params incomplete Structural image pre-processing missing |
|  | Tchistiakova, Crane, *et al.*, 2015 [75] | 4 | ✓ | ✓ | 🗶 | 🗶 "suspected of having WMH" | 🗶 | 🗶 | 🗶 | ✓ | 🗶 | ✓ | MRI acquisition params incomplete |
|  | Wu, Lai, *et al.*, 2015 [38] | 2 | 🗶 causal hypothesis vs. correlation analysis | 🗶 | 🗶 | 🗶 leukoaraiosis w/o lacunes | 🗶 | 🗶 | 🗶 | ✓ | 🗶 | 🗶 FWE correction method | ✓ |
|  | Yi, Liang, *et al.*, 2015 [51] | 1 | 🗶 causal hypothesis vs. correlation analysis | 🗶 | 🗶 | ✓ | 🗶 | 🗶 | 🗶 | 🗶 graph parameters | 🗶 | 🗶 FDR-method unclear | Handling of negative correlations |
|  | Kim, Cha, *et al.*, 2016 [57] | 5 | ✓ | ✓ | 🗶 | ✓ | 🗶 | 🗶 | 🗶 | ✓ | 🗶 | ✓ | MRI acquisition params incomplete  No information on confound regression |
|  | Zhou, Hu, *et al.*, 2016 [58] | 2 | 🗶 causal hypothesis vs. correlation analysis | 🗶 | 🗶 | ✓ | 🗶 | 🗶 | 🗶 | ✓ | 🗶 | 🗶 unclear how the cluster-forming threshold was determined  FDR-method unclear | MRI acquisition params incomplete |
|  | Cheng, Qi, *et al.*, 2017 [179] | 2 | 🗶Too broad: "investigate the cerebral function deficits" | ✓ | 🗶 | 🗶 "brain ischemic diseases" | 🗶 | 🗶 | 🗶 | ✓ | 🗶 | 🗶 multiple comparison correction unclear | MRI acquisition params incomplete  Global signal regression unclear |
|  | Ding, Ding, *et al.*, 2017 | 5 | ✓ | ✓ | 🗶 | ✓ | 🗶 | 🗶 | 🗶 | ✓ | 🗶 | 🗶 'outliers' unclear | ✓ |
|  | Li, Lai, *et al.*, 2017 [53] | 3 | ✓ | 🗶 No validation set | 🗶 | ✓ | 🗶 | 🗶 | 🗶 | ✓ Validity part of the study | 🗶 | 🗶 FDR-method unclear | not enough details |
|  | Ding, Ding, *et al.*, 2018 [48] | 3 | 🗶Exploratory vs confirmatory | ✓ | 🗶 | ✓ | 🗶 | 🗶 | 🗶 | ✓ | 🗶 | 🗶 FDR method unclear NBS threshold unclear | MRI acquisition params incomplete  Structural image pre-processing missing |
|  | Lawrence, Tozer, *et al.*, 2018 [35] | 6 | ✓ | ✓ | 🗶 | ✓ | 🗶 | 🗶 | 🗶 | ✓ | ✓ Reliability part of the study | ✓ | Missing details on confound regression and motion scrubbing |
|  | Chen, Li, *et al.*, 2019 [60] | 3 | 🗶 too broad | ✓ | 🗶 | ✓ | 🗶 | 🗶 | 🗶 | ✓ | 🗶 | 🗶 lack of information on covariates, multiple testing  FDR method unclear Handling of covariates | MRI acquisition params incomplete |
|  | Franzmeier, Rubinski, *et al.*, 2019 [39] | 7 | ✓ | ✓ | 🗶 | ✓ | ✓ | ✓ | 🗶 | ✓ | 🗶 | ✓ | MRI acquisition params incomplete |
|  | Liu, Chen, *et al.*, 2019 [34] | 4 | 🗶 Exploratory vs confirmatory | ✓ | 🗶 | ✓ | 🗶 | 🗶 | 🗶 | ✓ | 🗶 | 🗶 No details on mediation analysis | ✓ |
|  | Liu, Wu, *et al.*, 2019 [33] | 6 | ✓ | ✓ | 🗶 | ✓ | 🗶 | 🗶 | 🗶 | ✓ | 🗶 | ✓ | ✓ |
|  | Qin, Zhu, *et al.*, 2019 [37] | 5 | ✓ | ✓ | 🗶 | ✓ | 🗶 | 🗶 | 🗶 | 🗶 graph parameters | 🗶 | ✓ | ✓ |
|  | Wang, Chen, *et al.*, 2019 | 4 | ✓ | ✓ | 🗶 | ✓ | 🗶 | 🗶 | 🗶 | 🗶 graph parameters | 🗶 |  | Acquisitions params incomplete |
|  | Gesierich, Tuladhar, *et al.*, 2020 [50] | 6 | ✓ | ✓ | 🗶 | ✓ | 🗶 | 🗶 | 🗶 | 🗶 graph parameters | ✓ Reliability part of the study | ✓ | ✓ |
|  | Kumar, Vipin, *et al.*, 2020 [178] | 2 | 🗶 Too imprecise | 🗶 Reference group missing | 🗶 | ✓ | 🗶 | 🗶 | 🗶 | ✓ | 🗶 | 🗶 FDR-method unclear Multi-stage analysis not accounted for | MRI acquisition params incomplete |
|  | Shi, Chen, *et al.*, 2020 [182] | 2 | 🗶 Too broad: "reveal the pathogenesis of leukoaraiosis" | 🗶 | 🗶 | ✓ | 🗶 | 🗶 | 🗶 | ✓ | 🗶 | 🗶 FDR-method unclear | FDR-method unclear |
|  | Zhou, Zhang, *et al.*, 2020 [174] | 4 | 🗶 Exploratory vs confirmatory | ✓ | 🗶 | ✓ | 🗶 | 🗶 | 🗶 | ✓ | 🗶 | 🗶 Multi-stage analysis not accounted for | ✓ |
|  | Zhu, Lu, *et al.*, 2020 [40] | 0 | 🗶 Unclear if 'case-control' or observational study | 🗶 | 🗶 | 🗶 “Ischemic leukoaraiosis” | 🗶 | 🗶 | 🗶 | 🗶 graph parameters | 🗶 | 🗶 Handling of covariates | MRI acquisition params incomplete |

Table S2 Results of risk-of-bias assessments using the AXIS tool. Numbers in brackets refer to the reference list in the main manuscript file. Numbered columns 3—13 correspond to Items detailed in Table S1. Checks (✓) and crosses (🗶) indicate which Items are satisfied by each individual article. Risk of bias is computed as the sum of satisfied Items and shown in column 2.
